# Comparative metabarcoding and biodiversity of gut-associated fungal assemblages of *Dendroctonus* species (Curculionidae: Scolytinae)

**DOI:** 10.3389/fmicb.2024.1360488

**Published:** 2024-03-07

**Authors:** Rosa María Pineda-Mendoza, Jorge Luis Gutiérrez-Ávila, Kevin F. Salazar, Flor N. Rivera-Orduña, Thomas S. Davis, Gerardo Zúñiga

**Affiliations:** ^1^Laboratorio de Variación Biológica y Evolución, Departamento de Zoología, Escuela Nacional de Ciencias Biológicas, Instituto Politécnico Nacional, Mexico City, Mexico; ^2^Laboratorio de Ecología Microbiana, Departamento de Microbiología, Escuela Nacional de Ciencias Biológicas, Instituto Politécnico Nacional, Mexico City, Mexico; ^3^Department of Forest and Rangeland Stewardship, Warner College of Natural Resources, Colorado State University, Fort Collins, CO, United States

**Keywords:** bark beetles, symbiotes, core mycobiome, trophic mode, ITS2 region

## Abstract

The genus *Dendroctonus* is a Holarctic taxon composed of 21 nominal species; some of these species are well known in the world as disturbance agents of forest ecosystems. Under the bark of the host tree, these insects are involved in complex and dynamic associations with phoretic ectosymbiotic and endosymbiotic communities. Unlike filamentous fungi and bacteria, the ecological role of yeasts in the bark beetle holobiont is poorly understood, though yeasts were the first group to be recorded as microbial symbionts of these beetles. Our aim was characterize and compare the gut fungal assemblages associated to 14 species of *Dendroctonus* using the internal transcribed spacer 2 (ITS2) region. A total of 615,542 sequences were recovered yielding 248 fungal amplicon sequence variants (ASVs). The fungal diversity was represented by 4 phyla, 16 classes, 34 orders, 54 families, and 71 genera with different relative abundances among *Dendroctonus* species. The α-diversity consisted of 32 genera of yeasts and 39 genera of filamentous fungi. An analysis of β-diversity indicated differences in the composition of the gut fungal assemblages among bark beetle species, with differences in species and phylogenetic diversity. A common core mycobiome was recognized at the genus level, integrated mainly by *Candida* present in all bark beetles, *Nakazawaea*, *Cladosporium*, *Ogataea*, and *Yamadazyma*. The bipartite networks confirmed that these fungal genera showed a strong association between beetle species and dominant fungi, which are key to maintaining the structure and stability of the fungal community. The functional variation in the trophic structure was identified among libraries and species, with pathotroph-saprotroph-symbiotroph represented at the highest frequency, followed by saprotroph-symbiotroph, and saprotroph only. The overall network suggested that yeast and fungal ASVs in the gut of these beetles showed positive and negative associations among them. This study outlines a mycobiome associated with *Dendroctonus* nutrition and provides a starting point for future *in vitro* and omics approaches addressing potential ecological functions and interactions among fungal assemblages and beetle hosts.

## 1 Introduction

The genus *Dendroctonus* (Curculionidae: Scolytinae) is a Holarctic taxon composed of 21 nominal species, 19 of which are found in the Nearctic region from Alaska to Honduras and two in the Palearctic region in Europe and Asia (Victor and Zuniga, 2016; Valerio-Mendoza et al., 2019), although recently a North American taxon was indirectly introduced into China (Yan et al., 2005). Some of the species of these bark beetles are well-known disturbance agents of forest ecosystems, as their populations frequently experience outbreaks due to varying exogenous factors including droughts, warming climate, attributes of forest stands, topography, soil type, and humidity, resulting in the mortality of thousands to millions of trees with drastic effects on ecosystem structure, function, and composition (Kurz et al., 2008; Raffa et al., 2015).

The life cycle of *Dendroctonus* beetles consists of three phases: a short dispersal period, host tree colonization, and subcortical growth and development by new offspring. This last phase takes place mainly under the tree bark where colonizing adults and larvae feed on phloem tissue of the genera *Larix*, *Pseudotsuga*, *Picea*, and especially *Pinus* (Six and Bracewell, 2015). In the subcortical environment, *Dendroctonus* species are associated with many phoretic ectosymbiotic (e.g., filamentous fungi, yeasts and bacteria, nematodes, and mites) and endosymbiotic communities (e.g., yeasts and bacteria) (Hofstetter et al., 2015). These symbiotes are carried by beetles to trees on the external surface of their bodies, in specialized morphological structures, such as mycetangia and nematangia, and within the digestive system, including mouthparts and the alimentary canal (Beaver, 1989). Despite the strong association of *Dendroctonus* spp. with microbial communities, there is no evidence that they are acquired by vertical transmission, suggesting that they are acquired *de novo* in each new generation from the subcortical environment (Six and Bracewell, 2015; Hernández-García et al., 2017; Gonzalez-Escobedo et al., 2018; Vazquez-Ortiz et al., 2022).

Interactions between *Dendroctonus* species with their symbiotes have been studied intensively for many years (Klepzig and Six, 2004; Davis, 2015), even though these interactions are complex and dynamic in space and time (Six and Klepzig, 2021). The general pattern that emerges from earlier studies is that microbial associations are involved in several ecological processes that may enhance beetle fitness during the process of tree colonization and brood development. However, the interaction with specific members of microbial communities and their ecological role in many cases is not known. For example, some *Dendroctonus* are associated with both teleomorph and anamorph forms from several Ascomycetes (e.g., *Ophiostoma*/*Sporothrix*, *Ceratocystis*, *Ceratocystiopsis*, *Grosmannia*/*Leptographium*) and Basidiomycetes (e.g., *Entomocorticium*) (Paine et al., 1997; Harrington, 2005), which can affect the beetle lifecycle vis-à-vis obstruction of phloem and xylem tracheids, toxic terpenes detoxification, the regulation of insect development, the inhibition of pathogenic microorganisms, and nutritional supplementation (Bentz and Six, 2006; Davis et al., 2011; DiGuistini et al., 2011; Dai et al., 2022).

Bacteria are also common microbial associates of *Dendroctonus* spp., and *in vitro* studies show that the members of the genera *Arthrobacter*, *Bacillus*, *Brevundimonas*, *Cellulosimicrobium*, *Cellulomonas*, *Janibacter*, *Kocuria*, *Leifsonia*, *Methylobacterium*, *Paenibacillus*, *Ponticoccus*, *Pseudomonas*, *Pseudoxanthomonas*, *Rahnella*, *Serratia*, *Stenotrophomonas*, and *Sphingomonas* have abilities that match or complement many of the same ecological functions as fungi (Scott et al., 2008; Adams et al., 2009; Morales-Jiménez et al., 2009, 2012, 2013; Boone et al., 2013; Hu et al., 2014; Mason et al., 2014; Cano-Ramírez et al., 2016; Xu et al., 2016; Briones-Roblero et al., 2017a; Pineda-Mendoza et al., 2022).

Unlike filamentous fungi and bacteria, the ecological role of yeasts in the bark beetle is less well understood, although yeasts were the first group of microbes to be studied systematically in bark beetles (Davis, 2015). Several commonly isolated yeast species, including *Kuraishia capsulata*, *Ogataea pini*, *Nakazawaea holstii* (= *Pichia holstii*), *Wickerhamomyces bovis* (= *Pichia bovis*), *W. canadensis*, *Candida oregonensis*, *Cyberlindnera americana*, and *Zygoascus* sp., have abilities to degrade terpenes, starch, and lipids (Hunt and Borden, 1990; Briones-Roblero et al., 2017b), produce semiochemicals (Davis et al., 2023), or inhibit the growth of filamentous fungi (Davis et al., 2011). Many other yeasts have been isolated from mycetangia, gut, frass, and galleries of *Dendroctonus*, but their ecological functions have not been tested (Rivera et al., 2009; Lou et al., 2014; Dohet et al., 2016).

The above studies performed with cultivation-dependent methods demonstrated the functional capacities of specific members of assemblages, but they did not allow for an estimate of overall fungal biodiversity or phylogenetic diversity in beetle-microbe assemblages and, as a result, underestimated the diversity. They also did not elucidate the integral functional capacity of assemblages, or the importance of specific partners in them, as well as their relationships among one another. However, the advent of next-generation sequencing technologies has enabled more comprehensive and detailed analysis of *Dendroctonus* microbial communities, including the exclusiveness and persistence of microbes into communities, the analysis of the community structure across the life cycle of whole insects, geographic sites, beetle species, body parts, and field insects vs. laboratory rearing insects, as well as the identification of genes involved in terpene degradation (Adams et al., 2013; Durand et al., 2015, 2019; Dohet et al., 2016; Briones-Roblero et al., 2017b; Hernández-García et al., 2017, 2018).

Thus far, little is known about the gut mycobiome of *Dendroctonus* bark beetles (Rivera et al., 2009). Some studies have used culture-independent techniques to describe the fungal community composition and changes across the life cycle of whole insects (Lou et al., 2014; Dohet et al., 2016; Durand et al., 2019), with only one earlier study analyzing the composition of fungal assemblages in the mycetangia of *Dendroctonus frontalis* species complex (Vazquez-Ortiz et al., 2022). In this study, we aim to characterize and compare the gut-associated fungal community of 14 species of the genus *Dendroctonus* through the analysis of the internal transcribed spacer 2 (ITS2) region. Specifically, we describe how the community structure of the gut-associated fungal assemblages differs within and among species (α- and β-diversity) and examine whether a common mycobiome is shared or not among beetle species. Finally, we predict the functional ecological role of fungal assemblages and characterize interspecific linkages among fungi associates.

## 2 Materials and methods

### 2.1 Insect collection, dissection, and DNA extraction

During the summer of 2019, emerging adult insects of 14 species of *Dendroctonus* were collected directly from the galleries of infested pine and spruce trees in different localities from North and Central America (Table 1). Live insects were taken from galleries with sterile forceps and stored at 4°C in sterile polycarbonate Magenta^™^ vessels GA-7 (Sigma-Aldrich, United States) for transport to the laboratory. Insects collected in the United States were shipped inside sterile vials in absolute ethanol. All samples were processed immediately upon arrival at the laboratory. Taxonomic identification was verified according to the work of Armendáriz-Toledano and Zúñiga (2017).

**Table 1:** Collection localities of the 14 species of the *Dendroctonus* genus sampled in the present study.

Species	Locality	Latitude (°N)	Longitude (°W)	Altitude (m.a.s.l.)	Host tree
*D. adjunctus*	Nevado de Colima, Jalisco, Mexico	19°35′06.0″	103°36′14.4″	3,260	*Pinus hartwegii*
*D. approximatus*	San José Poaquil, Guatemala	14°49′30″	90°54′22″	1,527	*Pinus teocote/ Pinus montezumae*
*D. barberi*	Madera Canyon, Texas, USA	30°55′43″	103°48′49″	1,497	*Pinus* sp.
*D. brevicomis*	Rocheachi, Pesachi, Guachochi, Chihuahua, Mexico	27°4′55.91″	107°12′2.50″	2,800	*Pinus* sp.
*D. brevicomis*	Spring, Creek, Oregon, USA	42°40′46″	121°53′32″	1,725	*Pinus* sp.
*D. frontalis*	El Madroño, Querétaro, Mexico	21°16′49.2″	99°08′53.6″	1,687	*Pinus teocote*
*D*. *mesoamericanus*	Montebello Lagoon, Chiapas, Mexico	16°07′00″	91°42′00″	1,500	*Pinus oocarpa*
*D. mexicanus*	El Durazno, Guanajuato, Mexico	21°19′18.19″	99°47′5.494″	2,454	*Pinus teocote*
*D. parallelocollis*	km 10 Carr Uruapan, Michoacan, Mexico	19°24′23″	102°2′35″	1,615	*Pinus pringlei*
*D. ponderosae*	British Columbia, Canada	54°49′19.9″	124°44′36.4″	1,839	*Pinus* sp.
*D. rhizophagus*	San Juanito, Bocoyna; Chihuahua, Mexico	27°55′54.9″	27°35′54.6″	2,452	*Pinus arizonica*
*D. rufipennis*	Colorado, Washington, USA	39.03°75′62″	107.94°20′05″	3,657	*Picea engelmannii*
*D. terebrans*	Red Dirt National Wildlife, Kisatchie National Forest, Louisiana, USA	31°49′60.05″	93°25′59.24″	1,429	*Pinus* sp.
*D. valens*	Los pozos, Valentin Gómez Farias, Jalisco, Mexico	19°48′41′′	103°64′19′′	2,550	*Pinus montezumae*
*D. vitei*	El Cilantrillo, Nuevo León, Mexico	25°21′22.2″	100°19′32.7″	1,844	*Pinus cembroides*

All insects were surface disinfected by immersion in the following solutions at 1 min intervals: detergent solution (10 mmol L^−1^ Tris–HCl pH 8.0, 1 mmol L^−1^ EDTA, 10 mmol L^−1^ NaCl, 1% SDS, and 2% Triton X-100), 70% ethanol solution, and then washed three times with sterile distilled water. Afterward, they were placed in sterile phosphate buffer (PBS, pH 7.4; 137 mmol L^−1^ NaCl, 2.7 mmol L^−1^ KCl, 10 mmol L^−1^ NaHPO_4_, and 2 mmol L^−1^ KH_2_PO_4_) for dissection under aseptic conditions using a stereo microscope (MZ6 Leica, Germany). A longitudinal incision was performed with forceps scissors and sterilized fine-tipped to extract the gut, removing the elytra, wings, and tergites (Briones-Roblero et al., 2017b). We did not consider the sex of insects because previous studies performed with bacteria and yeasts through culture-independent and culture-dependent techniques did not show significant differences in sexes (Rivera et al., 2009; Briones-Roblero et al., 2017a). Three biological replicates per species were independently carried out, each replicate integrated by a pool of 10 guts. Homogenization of samples was performed in 1.5 mL Eppendorf tubes with 500 μL of sterile PBS and sterile plastic pestles. The metagenomic DNA was obtained with the DNeasy Blood & Tissue Kit (Qiagen, Valencia, CA, USA) according to the manufacturer’s specifications, and their concentration and purity were evaluated in a NanoDrop^™^ 2000c Spectrophotometer (Thermo Scientific, Wilmington DE, USA).

### 2.2 Sequencing and data analysis

The ITS2 amplification was carried out with the universal primer pairs ITS3 (5′-GCA TCG ATG AAG AAC GCA GC-3′) and ITS4 (5′-TCC TCC GCT TAT TGA TAT GC-3′). We used this region because it is less variable in size than ITS1, but sufficiently variable in nucleotide content to discriminate ASVs (Taylor et al., 2016). The amplicon libraries were sequenced using a paired-end 2 × 300 bp Illumina MiSeq sequencer according to the protocols of Macrogen Inc. (Seoul, Korea). The raw reads were deposited into the NCBI Sequence Read Archive (SRA) database (Bioproject number PRJNA1074716). Raw paired-end reads were processed in Quantitative Insights Into Microbial Ecology (QIIME2)^1^ v.2023.2, last accessed June 2023 (Bolyen et al., 2019). To remove low quality and/or uninformative features, the raw read sequences were subjected to a denoising process consisting of quality filtering (Phred 30 score), trimming, dereplication, merging, and the removal of chimera sequences using the DADA2 plugin (Callahan et al., 2016). To determine amplicon sequence variants (henceforth called ASVs) at a 97% identity threshold, we used the pre-trained Naive Bayes classifier against the UNITE database (Kõljalg et al., 2020).^2^ To confirm ASV taxonomic identity, they were manually verified in GenBank.^3^ ASV contaminants from insects, mites, nematodes, and plants were removed from the data set. ASVs were aligned with the Multiple Alignment using Fast Fourier Transform program (MAFFT, Katoh and Standley, 2013) to infer an unrooted maximum-likelihood phylogenetic tree with IQ-TREE (Nguyen et al., 2015) using as the best model of nucleotide evolution TIM2f + I + G4 *sensu* Akaike criterion (-InL = 22909.196, *I* = 0.12, and *G* = 0.925). Given the size of the libraries was uneven and the observed abundance data were compositional, the ASV abundances were standardized using the centered log-ratio (CLR) transformation in CoDaPack v.2.02.21 (Comas-Cufí and Thió-Henestrosa, 2011). As fungi taxonomy is dynamic and constantly changing, in this study, we employ the current taxonomic names and the lineage or clade name following those given in the NCBI database.

### 2.3 Diversity analysis of fungal assemblages

The sampling coverage was calculated using rarefaction curves and the Good’s coverage in QIIME2. ASV abundance at the phylum, class, order, family, and genus levels at 97% similarity was represented as bar plots using ggplot2 in the R v.4.2.1 package. The α-diversity of each fungal assemblage of *Dendroctonus* species was estimated with metrics Chao1 (species richness), Shannon-Weaver (diversity), Simpson and Simpson reciprocal (dominance) in QIIME2. To test for differences in α-diversity metrics, we used the Kruskal–Wallis test in QIIME2. Based on the presence or absence of fungal genera in all libraries, we inferred using UpSetR package v 1.4.0 (Conway et al., 2017) the total number of fungi, shared and unique, present in the gut of each beetle species, as well as a common mycobiome core (CMC) among them.

To infer the ability of some members representing the genera that were part of CMC, we downloaded genomes from NCBI^4^ and searched for genes involved in the degradation of fungal cell wall (chitin, glucan, and mannan) and plant cell wall (cellulose, hemicellulose, pectin, and lignin), as well as in the biosynthesis of essential amino acids. Gene prediction was carried out with AUGUSTUS v.2.5.5 (Stanke et al., 2004)^5^, and their functional annotation and metabolic pathways in which they participate delineated in the Kyoto Encyclopedia of Genes and Genomes (KEGG) database.^6^ In addition, the guild, the trophic mode, and the growth morphology of fungal ASVs in the gut of the *Dendroctonus* species were inferred using the FUNGuildR package (Nguyen et al., 2016)^7^, supplemented with information from the specialized literature.

β-diversity of gut fungal assemblages from *Dendroctonus* species was visualized using a principal component analysis (PCA) in scatterplot3d v.0.3–44 in the R package based on Aitchison distance (Aitchison, 1982; Aitchison et al., 2000), and weighted and unweighted UniFrac distances (Lozupone and Knight, 2005) using the CLR matrix in the phyloseq R package v.1.42.0 (McMurdie and Holmes, 2013). Significant differences in microbiome composition (β-diversity) between *Dendroctonus* species were performed by permutational multivariate analysis of variance (PERMANOVA) based on 9,999 random permutations in PAST v.4.10 (Hammer and Harper, 2001). In addition, to assess whether the composition of mycobiomes among within *Dendroctonus* species is similar, we performed the PERMDISP test with 10,000 permutations in QIIME2.

### 2.4 Co-occurrence analysis and nutritional and functional guilds

To determine the interactions among ASVs with ≥23 reads in each of the fungal assemblages of *Dendroctonus* species, a co-occurrence empirical network based on the random matrix theory was constructed using pairwise Spearman correlation (*p* < 0.05) in the molecular ecological network analysis (MENA) platform with default settings (Deng et al., 2012).^8^ To test whether the linkage among ASV interactions was due to non-random effects, a set of 100 random networks was generated from the empirical community structure. Significant differences between the empirical and the null random networks were evaluated using the Z test (*p* < 0.05). The random network was constructed with the Maslov-Sneppen method in MENA, which kept the number of nodes (taxa) and edges (connections) unchanged but rewired the positions of all links in the network. Network properties including modularity degree (M, a measure of the internal organization of the network into modules), average connectivity (avgK, average number of node connections within the network), average path distance (GD, average measure of the shortest paths between two nodes), and average clustering coefficient (avgCC, average measure of the extent to which nodes are grouped together in a network) were calculated in the MENA pipeline. The global network was visualized in the Cytoscape v.3.10.0 (Shannon et al., 2003). In addition, to understand the relative contribution of fungal genera to the structure and stability of the assemblages, we carried out a bipartite ecological analysis using the bipartite R package (Dormann et al., 2008). Two bipartite networks were built, the first with the most frequently occurring fungal genera and the second with the less abundant genera.

## 3 Results

### 3.1 Sequencing data

A total of 5,592,374 reads were obtained from 42 libraries from 14 *Dendroctonus* species. After quality control, 615,542 sequences were recovered, yielding a total of 248 fungal ASVs. Rarefaction curves and Good’s coverage (>99%) indicated that sampling effort for all libraries was appropriated to representatively capture taxonomic diversity (Supplementary Figure 1).

**Figure 1:**
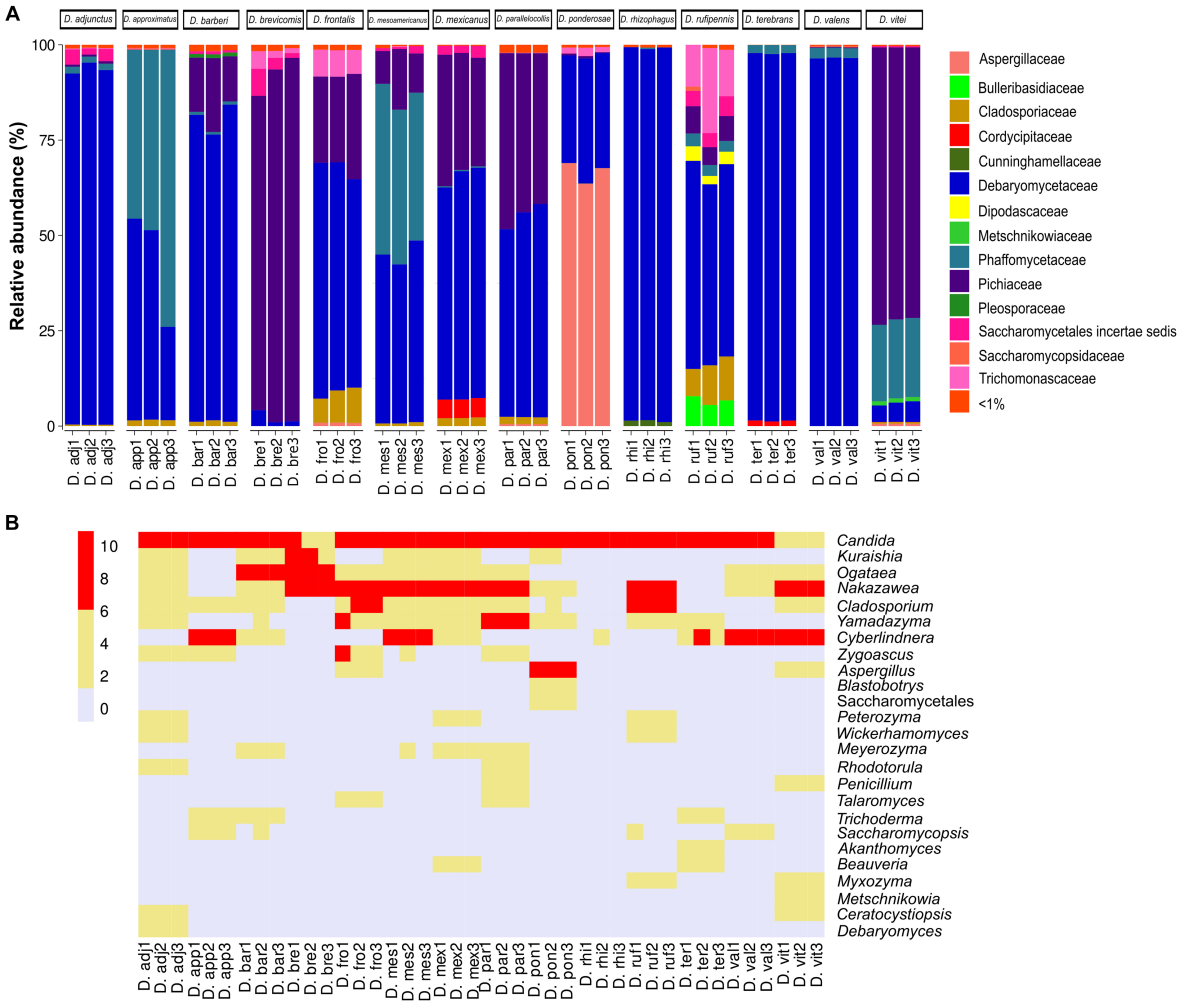
Fungal diversity and relative abundance of the gut assemblages associated with 14 species of the genus *Dendroctonus*. **(A)** A bar plot at the family level. **(B)** The heat map of the genera identified by bark beetle species. The color gradient shows the relative ASV’s abundance for each library.

The results showed a total of four phyla, 16 classes, 34 orders, 54 families, and 71 genera with different relative abundances among *Dendroctonus* species. The phylum most abundant was Ascomycota (99.85%), followed by Basidiomycota (0.13%), Mucoromycota (0.006%), and Mortierellomycota (0.002%). Specifically, the relative abundance of yeasts and fungi at the family and genus level varied among insect species (Figures 1A,B). Among the families that stand out as the most abundant are Debaryomycetaceae (49.7%), Pichiaceae (19.5%), Phaffomycetaceae (17.53%), Aspergillaceae (9.5%), and Cladosporiaceae (1.07%); the remaining families represented 2.7% of the relative abundance, but all of them had relative frequencies of <1% (Figure 1A).

In total, 32 genera of yeasts and 39 filamentous fungi were recorded, with the first being the more abundant than the second (Figure 1B), highlighting *Candida* (48.16%) as the most abundant in all *Dendroctonus* species, followed by *Nakazawaea* (18.13%), *Cyberlindnera* (17.44%), *Aspergillus* (9.44%), *Ogataea* (1.46%), *Yamadazyma* (1.42%), *Cladosporium* (1.07%), and other genera (2.88%) with <1% each of them of the total reads.

### 3.2 The analysis of fungal assemblage diversity

**Figure 2:**
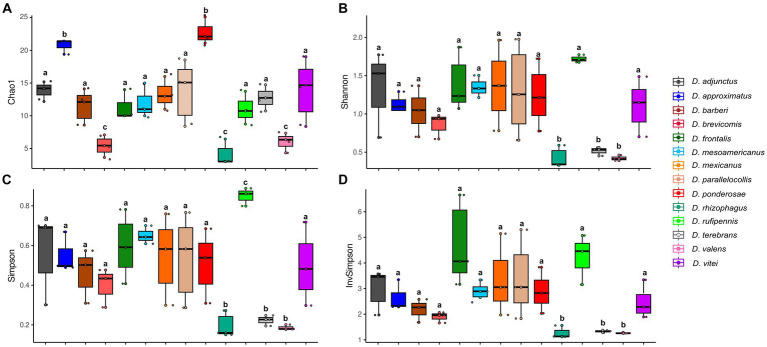
Alpha diversity indexes representing the fungal assemblage richness and diversity from 14 species of *Dendroctonus* genus. **(A)** Species richness by Chao1, **(B)** Shannon-Weaver diversity, **(C)** Simpson dominance, and **(D)** Simpson’s reciprocal diversity. Box plots not connected by the same letter are statistically different (*p* < 0.05).

According to Kruskal–Wallis and Dunn tests, the gut-associated fungal assemblages of *Dendroctonus* bark beetles showed statistically significant differences in species richness, species diversity, and dominance (*p* < 0.05; Figure 2). The higher species richness was found in assemblages of *D. ponderosae*, *D. approximatus*, and *D. vitei*, and the lowest species richness was found in those of *D. valens*, *D. brevicomis*, and *D. rhizophagus*. On the other hand, the gut fungal diversity in *D. rufipennis*, *D. frontalis*, and *D*. *mesoamericanus* assemblages was the most diverse with both metrics, whereas those from *D. rhizophagus*, *D. terebrans*, and *D. valens* were the least diverse. The number of dominant fungi varied among assemblages of bark beetle species from 2 to 5, with *D. valens* exhibiting the fewest dominant fungi in assemblages and *D. rufipennis* showing the most.

**Figure 3:**
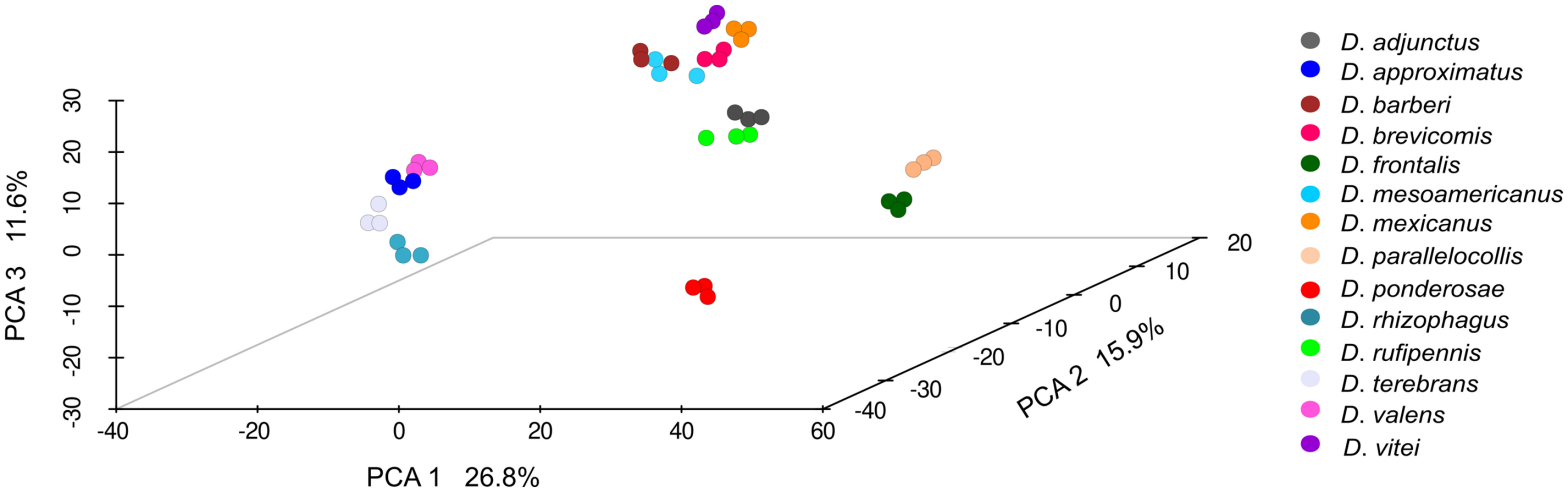
Principal component analysis (PCA, β-diversity) of the gut fungal assemblages from 14 species of *Dendroctonus*. (PERMANOVA, *p* < 0.05). Non-significant differences were found among within-species replicates (PERMDISP, *p* > 0.05). Dots of the same color represent biological replicates.

Statistically significant differences in the composition of the gut fungal assemblages (β-diversity) were found among bark beetle species according to the PERMANOVA tests using Aitchison distance (*F* = 51.704; *p* = 0.001) or unweighted (*F* = 45.072; *p* = 0.001) and weighted (*F* = 116.36; *p* = 0.001) UniFrac distances. The first three components of the PCA based on Aitchison distance explained 26.8, 15.9%, and 11.6% of the total variations observed among the bark beetle samples, respectively. The distribution of bark beetle assemblages in this multidimensional space showed a disjunct pattern among species, but no clear differentiation among within-species replicates (Figure 3). This pattern was also observed in the PCA using unweighted and weighted UniFrac distances but with different percentages of variation explained by the first three principal components (unweighted, C1 = 44.4%, C2 = 15.2%, and C3 = 10.1%; weighted, C1 = 70.1%, C2 = 13.2%, and C3 = 5.9%) (Plots shown in Supplementary Figures 2A,B). The PERMDISP test showed that dispersions around the mean or centroid were homogenous across samples (*F* = 7.72; *p* = 0.076).

**Figure 4:**
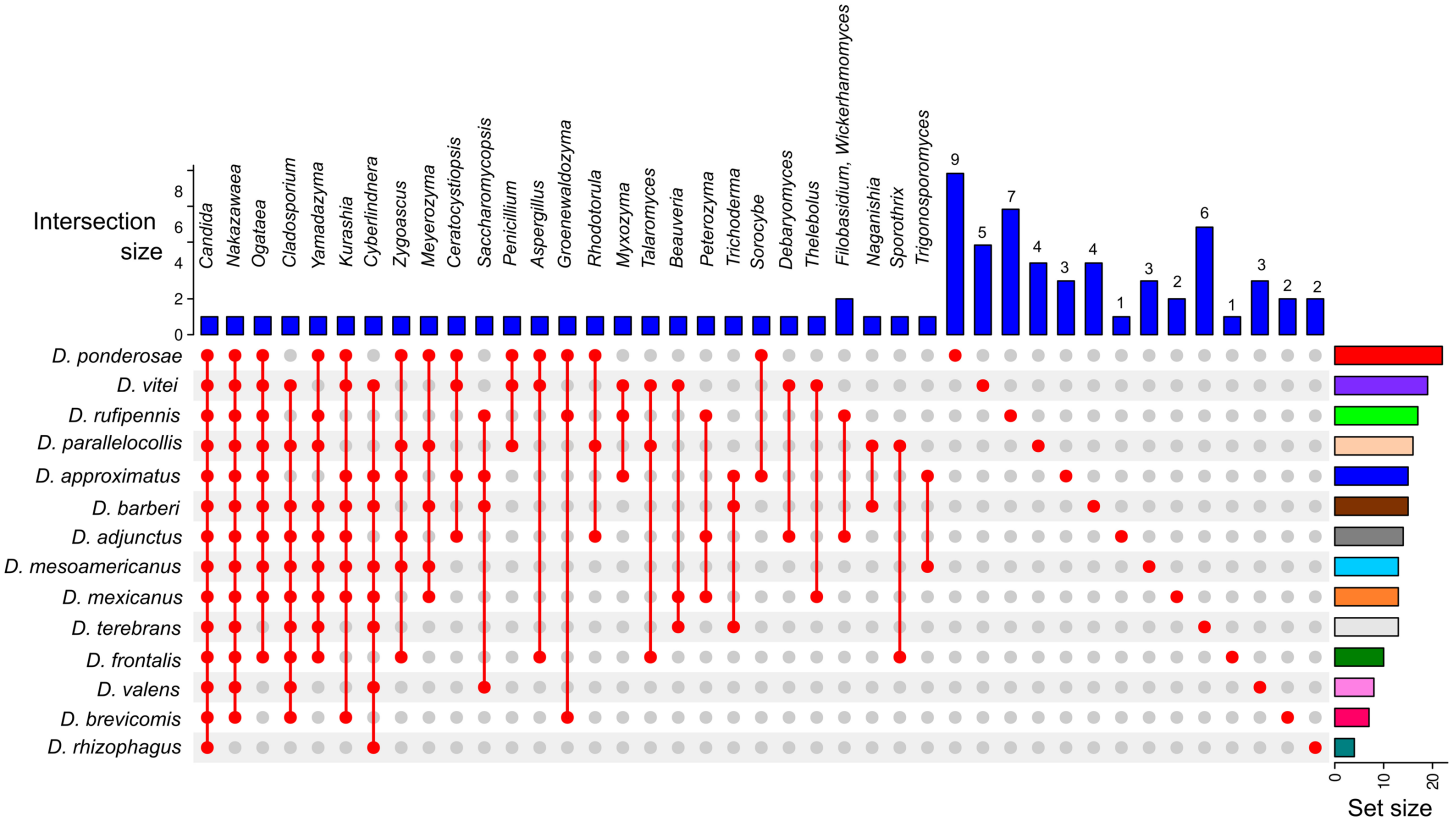
UpSetR plot of the core mycobiome from 14 *Dendroctonus* species. Colored bars (plot’s right side) highlight the number of total fungal genera present in each *Dendroctonus* species. Blue bars (plot’s top part) represent the number of fungal shared or single genera among beetle species. The red points (plot’s bottom) represent the presence or absence of fungal genera in the *Dendroctonus* species.

A relaxed CMC composed mainly of taxa in the genera *Candida* (present in all bark beetles, 100%), *Nakazawaea* (13 species; 92.86%), *Cladosporium* (10 species; 71.43%), *Ogataea* (10 species; 71.43%), and *Yamadazyma* (9 species; 64.28%) was recovered from the assemblages of *Dendroctonus* species (Figure 4). However, beetle species that shared the same fungal genera did not always share the same ASVs (Supplementary Table 1).

**Figure 5:**
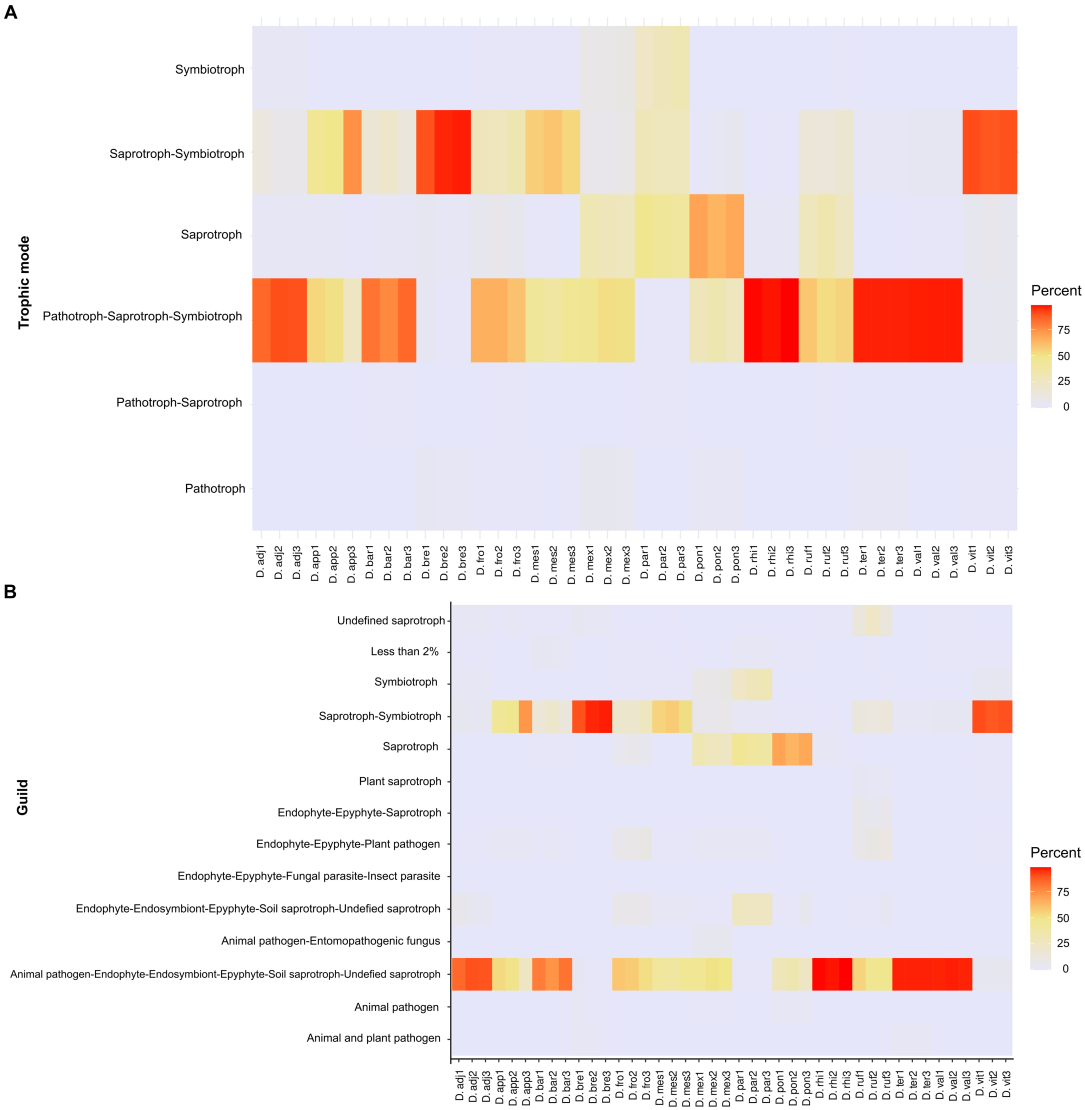
Heat maps showing relative frequencies of trophic modes **(A)** and guilds **(B)** predicted in each library of fungal assemblages, based on the FUNGuildR database.

An analysis of the metabolic pathways of the CMC (*Candida*, *Nakazawaea*, *Cladosporium*, *Ogataea*, and *Yamadazyma*) shows that these fungi do not possess all enzymes involved in the degradation of plant cell walls. Cellulose is degraded exclusively by *Cladosporium* (EC 3.2.1.4 and EC 3.2.1.21), whereas glucan (EC 3.2.1.58) is degraded by all five members, and there is no evidence that these fungi degraded lignin or rhamnose (Supplementary Figure 3). On the other hand, core fungi can synthesize threonine (EC 2.7.1.39 and EC 4.2.3.1) but do not have genes encoding the biosynthesis of essential amino acids (Ile, Val, Leu, Trp, Phe, Lys, His, and Met). A total of six trophic modes including 14 different guilds were identified from 248 fungal ASVs. The frequency of these trophic modes varied among libraries and beetle species, with the most frequent being pathotroph-saprotroph-symbiotroph (47.41%), saprotroph-symbiotroph (35.62%), and saprotroph only (14.31%), whereas the less frequent was symbiotroph (1.48%), and other trophic modes had a frequency of less than 2%. ASV into these trophic modes were associated to animal pathogen, endophyte, endosymbiont, epiphyte, saprotroph, undefined saprotroph, soil and wood saprotroph, as well as animal or plant symbiotroph (Figure 5; Supplementary Table 2).

### 3.3 Network analysis of fungal assemblages

**Figure 6:**
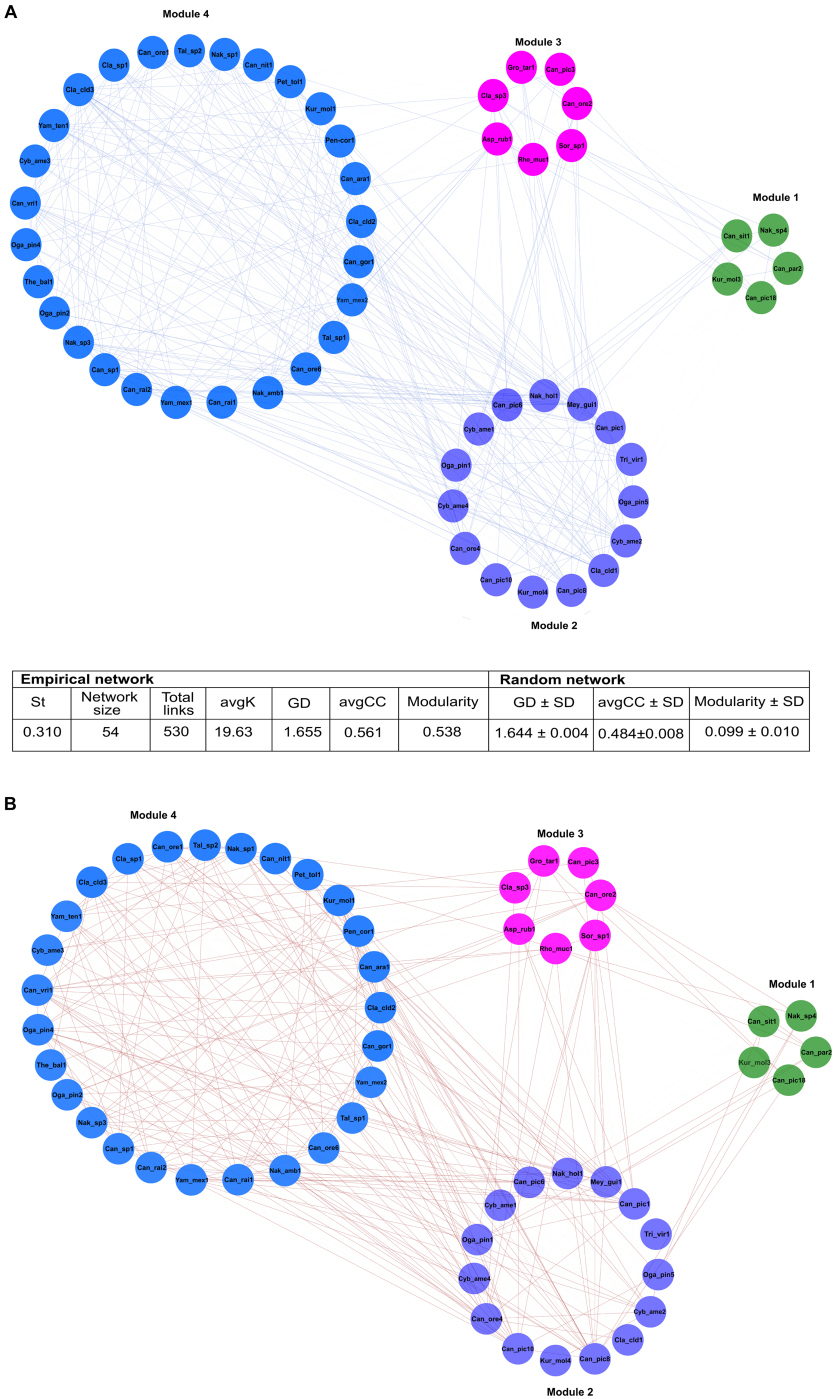
The general ecological networks of the fungal assemblages associated with 14 species of *Dendroctonus* genus. It was built with 115 ASVs whose reads were > 23. Four modules integrated in the general network, which included 11 ASV nodes from 7 genera of filamentous fungi, and 43 ASV nodes from 10 yeasts genera. The values of modularity, average connectivity, average path distance, and average clustering coefficient were significantly higher than the random network. Significant positive **(A)** or negative **(B)** correlations (*p* < 0.05) within the same and different modules were separated and represented by blue and red lines, respectively. Asp_rub (*Aspergillus ruber*); Can (*Candida* sp.); Can_ara (*Candida arabinofermentans*); Can_gor (*Candida gorgasii*); Can_nit (*Candida nitratophila*); Can_ore (*C. oregonensis*); Can_par (*C. parapsilosis*); Can_pic (*C. piceae*); Can_rai (*C*. *railenensis*); Can_sit (*Candida sithepensis*); Can_vri (*C*. *vriesea*); Cla (*Cladosporium* sp.); Cla_cld (*C*. *cladosporioides*); Cyb_ame (*Cyberlindnera americana*); Kur_mol (*Kuraishia molischiana*); Gro_tar (*Groenewaldozyma tartarivorans*); Mey_gui (*Meyerozyma guilliermondii*); Nak_sp. (*Nakazawaea*_sp.); Nak_amb (*N. ambrosiae*); Nak_hol (*N*. *holstii*); Oga_pin (*Ogataea pini*); Pen_cor (*Penicillium corylophilum*); Pet_tol (*Peterozyma toletana*); Rho_muc (*Rhodotorula mucilaginosa*); Sor_sp (*Sorocybe* sp.); Tal_sp (*Talaromyces* sp.); The_bal (*Thelebolus balaustiformis*); Tri_vir (*Trichoderma viride*); Yam_mex (*Yamadazyma mexicana*); and Yam_ten (*Yamadazyma tenuis*).

From 248 fungal ASVs recovered, only 115 had >23 reads. The global network was integrated by four modules, which included 11 ASV nodes from seven filamentous fungi, and 43 ASV nodes from 10 yeasts (Figure 6). The 54 nodes were connected by 530 statistically significant edges, of which 291 were positive edges (i.e., mutualistic and communalistic interactions) and 239 were negative edges (i.e., antagonistic interactions) (Figures 6A,B). In the global network, there was no presence of hub nodes (nodes that have a low within-module connectivity, zi < 2.5, Pi > 0.3); however, nine of them were connectors (nodes with a high fraction of their links to other modules, zi < 2.5, Pi ≥ 0.62) and 45 peripherals (nodes with most links within their module and with other modules, zi < 2.5; Pi < 0.62). The ASV *Candida* Can_ore2 was the no-hub peripheral node with the highest level of edges, and the ASV *Nakazawaea* Nak_amb1 was the connector no-hub node with the highest connectivity and efficiency among all ASV interactions in the network. The global network was statistically significant to null random networks (z-score = 2.75, *p* < 0.003), and the connectivity distribution followed the power law model (*R*2 = 0.001). The values of modularity (*M* = 0.538), average connectivity (avgK = 19.63), average path distance (GD = 1.655), and average clustering coefficient (avgCC = 0.561) were significantly higher in the global network than the null random network values (*M* = 0.099 ± 0.010, GD = 1.644 ± 0.004, and avgCC = 0.484 ± 0.008).

Module 1 comprised five ASVs in total: three of *Candida* (Can_par2, Can_sit1, Can_pic18), one of *Nakazawaea* (Nak_sp4), and one of *Kuraishia* (Kur_mol3) from four beetle species. These ASVs presented five positive and two negative interactions among them. Can_sit1 and Kur_mol3 ASVs connected module 1 with module 2, and Can_pic18, Can_par2, and Nak_sp4 with modules 2 and 3 (Supplementary Table 2).

Module 2 was formed by 15 ASVs in total: five of *Candida* (Can_ore4, Can_pic8, Can_pic10, Can_pic6, Can_pic1), one of *Meyerozyma* (Mey_gui1), two of *Ogataea* (Oga_pin1, Oga_pin5), three of *Cyberlindnera* (Cyb_ame1, Cyb_ame4, Cyb_ame2), one of *Kuraishia* (Kur_mol4), one of *Nakazawaea* (Nak_hol1), and two filamentous fungi of the genera *Trigonosporomyces* (Tri_vir1) and *Cladosporium* (Cla_cld1) from 13 beetle species. Within this module, ASVs had 43 positive and 14 negative interactions among them. Can_pic1, Can_pic10, Can_ore4, Cyb_ame2, and Cla_cld1 linked module 2 with modules 3 and 4; Can_pic1, Can_pic6, Can_pic8, Cyb_ame1, Oga_pin1, Nak_hol1, and Mey_gui1 linked it to modules 1, 3, and 4; Oga_pin5 connected it to modules 1 and 3; Kur_mol4 linked it to the modules 0 and 3; and Tri_vir1 associated it with the modules 2 and 3 (Supplementary Table 3).

ASVs of the yeasts *Candida* (Can_ore2 and Can_pic3), *Groenewaldozyma* (Gro_tar1), and *Rhodotorula* (Rho_muc1), and the filamentous fungi, *Cladosporium* (Cla_sp3), *Aspergillus* (Asp_rub1), and *Sorocybe* (Sor_sp1) from eight beetle species comprised module 3. These ASVs presented eight negative and 10 positive links among them within the module. Can_ore2, Can_pic3, Rho_muc1, Asp_rub1, and Cla_sp3 interconnected this module with the modules 1, 2, and 4, while Gro_tar1 and *Sorocybe* (Sor_sp1) linked it with the modules 1 and 2 (Supplementary Table 2).

Module 4 consisted of 23 ASVs of the yeasts *Nakazawaea* (Nak_sp1, Nak_amb1, and Nak_sp3), *Candida* (Can_ore1, Can_ore6, Can_rai1, Can_rai2, Can_sp1, Can_vri1, Can_gor1, Can_nit1, and Can_ara1), *Peterozyma* (Pet_tol1), *Kuraishia* (Kur_mol1), *Yamadazyma* (Yam_mex1, Yam_mex2, and Yam_ten1), *Ogataea* (Oga_pin2 and Oga_pin4), and *Cyberlindnera* (Cyb_ame3) and seven ASVs from the filamentous fungi *Cladosporium* (Cla_sp1, Cla_cld2, Cla_cld3), *Penicillium* (Pen_cor1), *Talaromyces* (Tal_sp1 and Tal_sp2), and *Thelebolus* (The_bal1) from 10 beetle species. These ASVs had 86 negative and 96 positive links between them within the module. Most of the ASVs were linked to modules 2 and 3, while Can_rai2, Can_gor1, Can_ore1, Can_ore1, Can_ore6, Can_nit1, Can_sp1, Yam_mex1, and Cla_sp1 linked it to module 2; Oga_pin2 connected it to module 2; Can_rai1 linked it to modules 1, 2, and 3; and Can_rai1 linked it to the modules 1, 2, and 3 (Supplementary Table 3).

**Figure 7:**
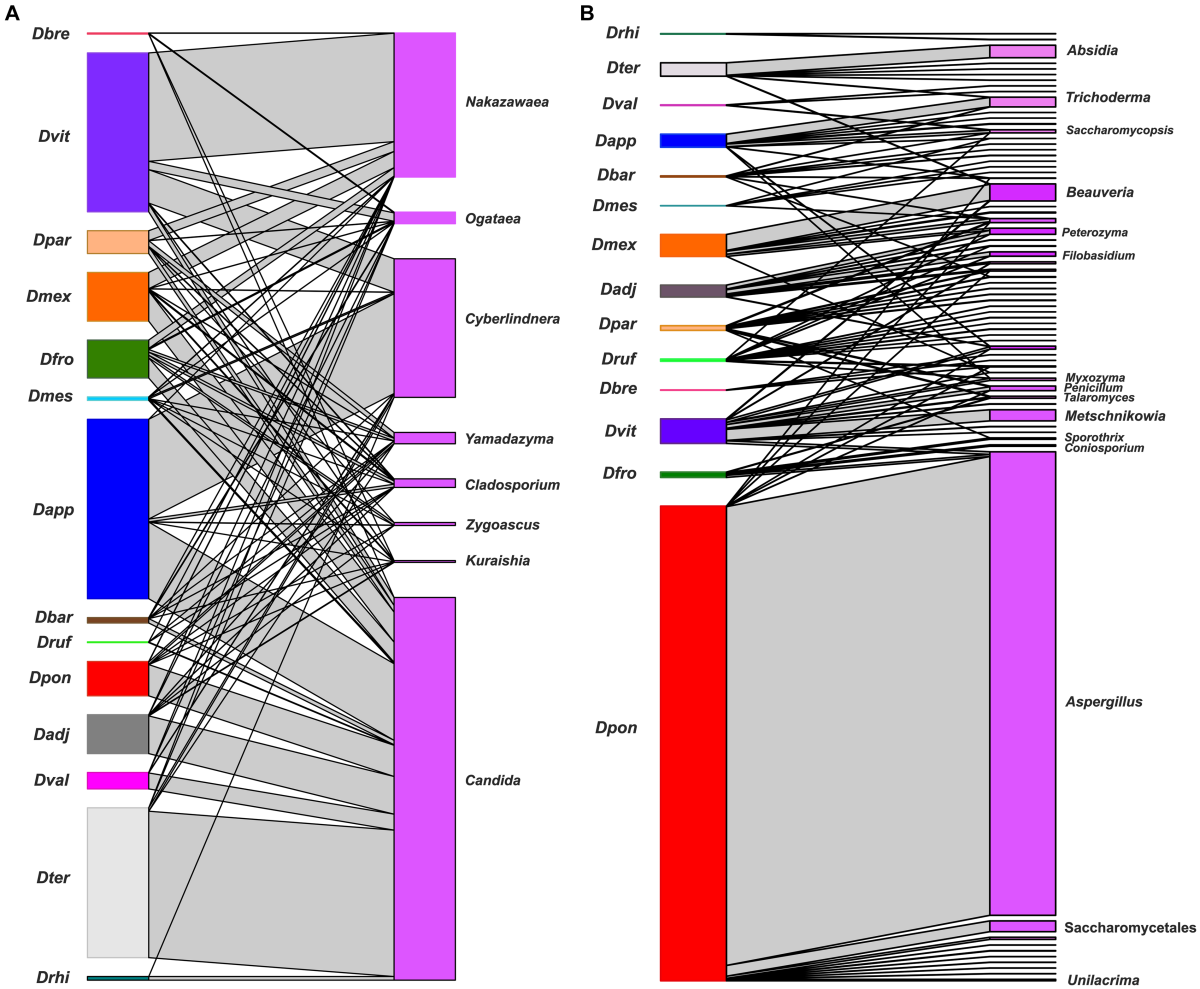
A bipartite network between *Dendroctonus* species and the gut fungal assemblages. *Dendroctonus* species are represented in the nodes on the left side and fungal ASVs in the nodes on the right side, the connections between insects and fungi are represented by gray connectors (or links). **(A)** The network of the most dominant fungal genera. **(B)** The network of non-dominant fungal genera. Dadj: *D. adjunctus*, Dapp: *D. approximatus*, Dbar: *D. barberi*, Dbre: *D. brevicomis*, Dfro: *D. frontalis*, Dmes: *D*. *mesoamericanus*, Dmex: *D. mexicanus*, Dpar: *D. parallelocollis*, Dpon: *D. ponderosae*, Drhi: *D. rhizophagus*, Druf: *D. rufipennis*, Dter: *D. terebrans*, Dval: *D. valens*, and Dvit: *D. vitei*.

The bipartite networks confirmed that *Candida*, *Cyberlindnera*, *Yamadazyma*, *Cladosporium*, *Ogataea*, and *Nakazawaea* were key genera that maintained the structure and stability of the community (modularity 0.38, nestedness 19.0, and connectedness 0. 65). However, a plethora of fungal genera of low frequency showed some degree of specialization in each beetle assemblages (modularity 0.27, nestedness 26.07, and connectedness 0.10) (Figure 7).

## 4 Discussion

In this study, we comprehensively characterized the composition and diversity of gut-associated fungi of *Dendroctonus* bark beetles using the ITS2 region and high-throughput sequencing technologies. Our results indicate that gut fungal assemblages of the *Dendroctonus* spp. are represented mainly by Ascomycetous fungi, which agrees with other reports from the bark and ambrosia beetles, as well as other Coleoptera groups (Harrington, 2005; Suh et al., 2005; Henriques et al., 2006; Rivera et al., 2009; Gao et al., 2018; Biedermann and Vega, 2020; Chakraborty et al., 2020, 2023; Ibarra-Juarez et al., 2020; Veselská et al., 2023).

At the genus level, α-diversity was integrated into 71 fungal genera including 32 yeast ASVs and 39 filamentous fungi ASVs. Differences in richness and diversity among *Dendroctonus* gut assemblages were driven mainly by filamentous ASVs from fungi and yeast with a relative abundance of <1%. However, our results confirm that yeasts are the most abundant group and persistent in the gut of these bark beetles, which matches earlier reports for *Dendroctonus* spp. using culture-dependent methods (Rivera et al., 2009) and with other studies carried out with bark beetles that find yeasts and yeast-like fungi are primary taxa in gut assemblages (e.g., Chakraborty et al., 2020).

Gut fungal assemblages of *Dendroctonus* beetles differed considerably in α-diversity, including taxonomic ASV richness and abundance. In general, *Dendroctonus* species that feed on roots or the lower bole or colonize trees non-lethally in their native ranges, such as *D. rhizophagus*, *D. terebrans*, and *D. valens*, exhibited the least diverse gut fungal assemblages (Figure 2). In contrast, highly “aggressive” or “primary” *Dendroctonus* spp. that are often a driving factor in tree death, including *D. adjunctus*, *D. frontalis*, *D*. *mesoamericanus*, and *D. rufipennis*, generally had more diverse gut fungal assemblages. However, metrics of α-diversity also varied strongly across biological replicates for some species (e.g., *D. mexicanus*, *D. parallelocollis*, and *D. vitei*), indicating a potential need for additional sampling within species or across regions to fully characterize community variation.

The dominant yeasts were *Candida*, *Nakazawaea*, *Cyberlindnera*, *Yamadazyma*, and *Ogataea* (Figure 1). The high prevalence of yeast in the gut indicates that they are potentially important for beetle development and nutrition. Two pathways exist by which yeasts may impact beetle nutrition. First, during development, beetles almost certainly directly consume yeast cells that proliferate in the subcortical gallery environment. To the best of our knowledge, no studies have yet tested variation in beetle development with and without access to gut yeasts, likely due to the difficulties in rearing *Dendroctonus* axenically. One recent study showed that crude P content, but not N content, of yeast cells was not strongly different from tree phloem (Davis et al., 2023), indicating that at least some yeast isolates are not substantially more protein-rich than tree tissues alone. However, yeasts also produce enzymes that may be involved in the degradation of structural and non-structural polysaccharide complexes including cellulose, lignin, or starch, which could alter the C:N ratios or reduce tissue toughness. For instance, *Candida arabinofermentans* converts L-arabinose, a common pentose in lignocellulose, to ethanol (Kurtzman and Dien, 1998); *Candida nitratophila* assimilate nitrates (Ali and Hipkin, 1986); and both *Cyberlindnera americana* and *Candida piceae* have lipolytic and amylolytic abilities (Briones-Roblero et al., 2017b).

Second, yeasts may alter nutritional opportunities indirectly via community-driven effects. For example, some yeasts (e.g., *Ogataea*) can alter the growth of filamentous fungal symbionts (Paine and Hanlon, 1994; Davis et al., 2011), while others (e.g., *Yamadazyma*) in some cases inhibit it (Wang et al., 2013; Davis et al., 2023; González-Gutiérrez et al., 2023). In addition, some yeast taxa (e.g., *Kuraishia*, *Ogataea*, and *Nakazawaea*) degrade terpenes and/or affect pheromone production (Hunt and Borden, 1990), and several *in silico* studies in yeasts (e.g., *Candida* and *Cyberlindnera*) demonstrate the presence of genes and transcripts related to terpene detoxification, such as cytochromes P450, and Transporters ABC, MATE, and MFS, and others (Hernández-Martínez et al., 2016; Soto-Robles et al., 2019).

The low abundance recorded in the gut of the fungal symbionts, including *Ceratocystiopsis*, *Entomocorticium*, *Ophiostoma*/*Sporothrix*, and *Grosmannia*/*Leptographium,* is surprising and consistent with other culture-based and culture-independent studies (Rivera et al., 2009; Bozorov et al., 2019), confirming that these symbionts are primarily associated with mycetangia (Vazquez-Ortiz et al., 2022) or body surfaces (Gao et al., 2018; Chakraborty et al., 2020) from *Dendroctonus* bark beetles. It is generally regarded as canon that *Dendroctonus* beetles feed on their respective associated mycetangial fungal taxa during development (Six and Klepzig, 2004; Six and Wingfield, 2011); however, it is puzzling why these taxa (*Entomocorticium*, *Ophiostoma*, *Grosmannia*) were not abundant in the gut if this is the primary nature of the association.

There are several possible explanations for this pattern. First, insect digestive tracts contain microenvironmental variation in oxic and anoxic gradients (Ceja-Navarro et al., 2014), which may limit the proliferation of mycetangial fungi in the gut. Second, mycetangial fungi may be rapidly digested by beetles or degraded by joint action of chitinases present in both bacteria and yeasts (Fabryová et al., 2018; Bozorov et al., 2019; Ibarra-Juarez et al., 2020; Peral-Aranega et al., 2023). Third, presumably mycetangial fungi are fed upon primarily during larval development, which would lead us to assume that they should be present at this stage of development. However, these filamentous fungi are not isolated from the gut of *Dendroctonus* larvae using culture-dependent methods (Rivera et al., 2009). Even so, more detailed studies are required to confirm that larvae may have abundances of very different gut fungal assemblages than adult *Dendroctonus*, because individuals sampled in the present study were adults.

Instead, the dominant filamentous fungus in the alimentary canal was *Cladosporium* (present in 71% of beetle species), with approximately equivalent representation across species of *Aspergillus*, *Beauveria*, *Ceratocystiopsis*, and *Trichoderma* (present in ~21–28% of beetle species). It is unclear which of these associations are truly “symbiotic” relationships and which are incidental or even exploitative. *Beauveria* is an important entomopathogen for bark beetles and insects in general (Mann and Davis, 2021); similarly, some species of *Aspergillus* are pathogenic to bark beetles (Zeiri et al., 2017), though both fungi are relatively ubiquitous in natural environments (Ramirez-Camejo et al., 2012; Maistrou et al., 2020). Their presence in the gut of several species may indicate potential fungal parasitism in sampled populations. The ecological relationships between *Cladosporium* spp. and bark beetles are less clear; though some *Cladosporium* isolates may elevate the N content of plant tissues (Solomon and Oliver, 2001) or participate in the lignin degradation by the presence of laccases (Aslam et al., 2012). *Cladosporium* is isolated from various bark beetle species in many different geographic regions (Bateman et al., 2016; Davydenko et al., 2017). *Cladosporium* spp. are also environmentally ubiquitous on plant surfaces (Katsoula et al., 2021) but may also be endophytes (Ganley and Newcombe, 2006), plant pathogens (De Wit et al., 1997), entomopathogens (Samways and Grech, 1986), and even parasites of other fungi (Torres et al., 2017). Since *Cladosporium* spp. are represented in the gut of >70% of *Dendroctonus* species, further exploration of their role in nutrition and interactions with beetles and host trees is warranted.

On the other hand, results show a different β-diversity when guts among beetle species were compared (Figure 3; Supplementary Figures 2A,B), without the influence of the intraspecific variation of assemblages of each insect species. Different metrics indicate that each beetle species was generally associated with a relatively distinct gut fungal assemblage. The spatial disjunction of fungal assemblages (PCA) indicates a strong replacement of fungal ASVs, though there was also overlap in assemblages across species (Figure 4; Supplementary Figures 2A,B). Differences are given both by fungal ASVs with high and low abundance of shared genera between assemblages, as well as by unique or rare ASVs commonly of low abundance.

Although *Dendroctonus* spp. surveyed here were associated fully with the genus *Candida* and most with *Nakazawaea*, *Ogataea*, and *Yamadazyma*, our results show a core mycobiome integrated by different *Candida* ASVs (Figures 4, 5), which significantly contribute to the structure and stability of the community. This also suggests that beetles are often associated with cryptically diverse and functionally redundant assemblages of gut symbionts with potentially complementary enzymatic capabilities, to the benefit of all, including the insect host. Furthermore, this also suggests that the maintenance of fundamental functions across the incorporation or retention of symbionts with complementary capabilities or redundant is a viable strategy, which favors all interaction types among fungal symbionts as suggested by our ecological and bipartite networks, rather than the retention of strict specific symbionts.

The observed differences in both diversities may be due to different factors, such as biological (e.g., the type of life cycle and its duration and generation number), ecological (e.g., tree host species, distribution range both of insects and host, colonization strategy, endemic or epidemic condition of populations, and interaction with phoretic ectosymbionts or with other arthropods) and morpho-functional (e.g., gut compartmentalization and physicochemical microenvironment), which are dynamic in space and time and non-mutually exclusive. Furthermore, some of these factors can be influenced by the diet and the subcortical environment (Romeralo et al., 2022; Chakraborty et al., 2023; Ge et al., 2023), despite these insects exploiting a similar resource in terms of nutrition and habitat. Given that we lack a general understanding of how these factors affect mycobiome diversity (Rivera et al., 2009; Davis, 2015; Gao et al., 2018), in part because it is regulated by overlapping processes on spatial and temporal scales ranging from the global to the local, future studies with specific experimental designs are required to test the individual influence of different drivers.

The multiple positive or negative associations observed in the overall network suggest that fungal ASVs establish intimate interactions among them, which may be cooperative or mutualistic, as they contribute to the processes of terpene detoxification, degradation of complex polysaccharides, or nutrient recycling. On the contrary, negative interactions are surely related to the regulation and stabilization of a minimal community rather than to the phenomena of competition or repulsion among them, because the gut is a highly selective habitat, metabolically dynamic, and spatially limiting by its compartmentalization and microenvironmental condition changing and dynamics during the development of these insects. The presence of a common mycobiome integrated only by the genus *Candida* and the replacement of ASVs suggest the integration of a functional, not taxonomic (Risely, 2020) whose members are acquired from the environment, as has been suggested in other studies (Hernández-García et al., 2018; Vazquez-Ortiz et al., 2022). However, regardless of the ecological interaction of fungal members within *Dendroctonus* spp. gut assemblages, the trophic mode of assemblages may change depending on the trade-offs faced by the beetles during their life cycle, which undoubtedly affects the community structure and stability, as well as interactions among its members.

Several limitations to our study should be considered when interpreting the results reported here. First, ITS2 has multiple copies in the genome of some fungal species, and it must be acknowledged that as a result, some taxa may have inflated estimates of ASV abundance (Lavrinienko et al., 2021). Accordingly, ASV abundance may not directly translate to the abundance of fungal taxa as represented by biomass or colony forming units. However, various studies, including other studies of bark beetle microbial associations, have used this approach to describe general patterns within and between ecological communities (e.g., Chakraborty et al., 2020; Vazquez-Ortiz et al., 2022). In addition, although we detected correlations in ASV abundances, it is not clear whether these correlations are due to interactions within the observed microbial community or some other unmeasured factor.

Here, we report apparently functionally redundant fungal assemblages associated with the gut of *Dendroctonus* bark beetles, with a core mycobiome consisting of *Candida* ASVs, which were not the same among beetle assemblages. The α- and β-diversity observed among beetle species assemblages were relatively different and overlapping. Yeasts dominated the communities, although several filamentous fungi were also common across beetle taxa and warrant additional ecological study (e.g., *Cladosporium* spp.). It is not clear whether observed gut assemblages vary generationally or remain somewhat constant over time in populations, but this will be important to investigate to determine whether gut communities are reassembled *de novo* for each beetle generation. There are likely a variety of biological, ecological, and physiological factors involved in the integration of *Dendrocton*us gut assemblages; however, their role in providing nutrition to beetles has not yet been clearly determined.

Furthermore, a high degree of complexity in the global beetle-fungal network was observed, which would be expected to increase greatly in the presence of other beetle-associated microorganisms such as bacteria and bacteriophages. However, distinct modules of correlated fungal taxa were also observed, and functional differences among these modules have not yet been described but may be considerable. Understanding patterns of nutrient acquisition is a key area of investigation in insect ecology (e.g., Chandler et al., 2008; Behmer, 2009), and the nitrogen budget of phloem-feeding bark beetles remains difficult to explain without considering fungal symbioses (Ayres et al., 2000). In fact, a comparison between recent studies of mycetangial fungi diversity in *Dendroctonus* (Vazquez-Ortiz et al., 2022) and the present study clearly shows that fungal communities strongly differ between secretory fungal transport structures (mycetangia) and intestinal tracts. Future studies using omics techniques combined with *in vitro* or *in vivo* experiments will contribute to understanding the metabolic capacities of dominant members of the gut fungal assemblage, their forms of interaction, and their contributions to the survival and adaptation of tree-killing insects.

## Data availability statement

The datasets presented in this study can be found in online repositories. The names of the repository/repositories and accession number(s) can be found in the article/Supplementary material.

## Author contributions

This work was conceived by FR-O and GZ. Experiments were performed by JG-Á. The results were interpreted by RP-M, JG-Á, KS, FR-O, TD, and GZ. Drafts and final edition of the manuscript were written by RP-M, JG-Á, KS, FR-O, TD, and GZ. All authors contributed and approved the final manuscript.

## Footnotes

^1^https://qiime2.org/

^2^http://unite.ut.ee, accessed June 2023.

^3^https://blast.ncbi.nlm.gov/Blast.cgi

^4^https://www.ncbi.nlm.nih.gov/

^5^https://bioinf.uni-greifswald.de/augustus/

^6^https://www.genome.jp/kegg/

^7^https://github.com/UMNFuN/funguild

^8^http://ieg4.rccc.ou.edu/mena/login.cgi; accessed April 2023.
