# Supplementary material for: Comparative metabarcoding and biodiversity of gut-associated fungal assemblages of Dendroctonus species (Curculionidae: Scolytinae)
Source: Front Microbiol. 2024 Mar 7;15:1360488. doi: 10.3389/fmicb.2024.1360488 (PMC10959539; doi:10.3389/fmicb.2024.1360488)
Supplement: Supplementary file 4 [file Data_Sheet_1.DOCX]

**Supplementary Material**

**Comparative metabarcoding and biodiversity of gut-associated fungal assemblages of *Dendroctonus* species (Curculionidae: Scolytinae)**

**Rosa María Pineda-Mendoza^1^, Jorge Luis Gutiérrez-Ávila, Kevin F. Salazar^1^, Flor N. Rivera-Orduña^2^, Thomas S. Davis^3^ and Gerardo Zúñiga^1*^**

# Supplementary Figures

**
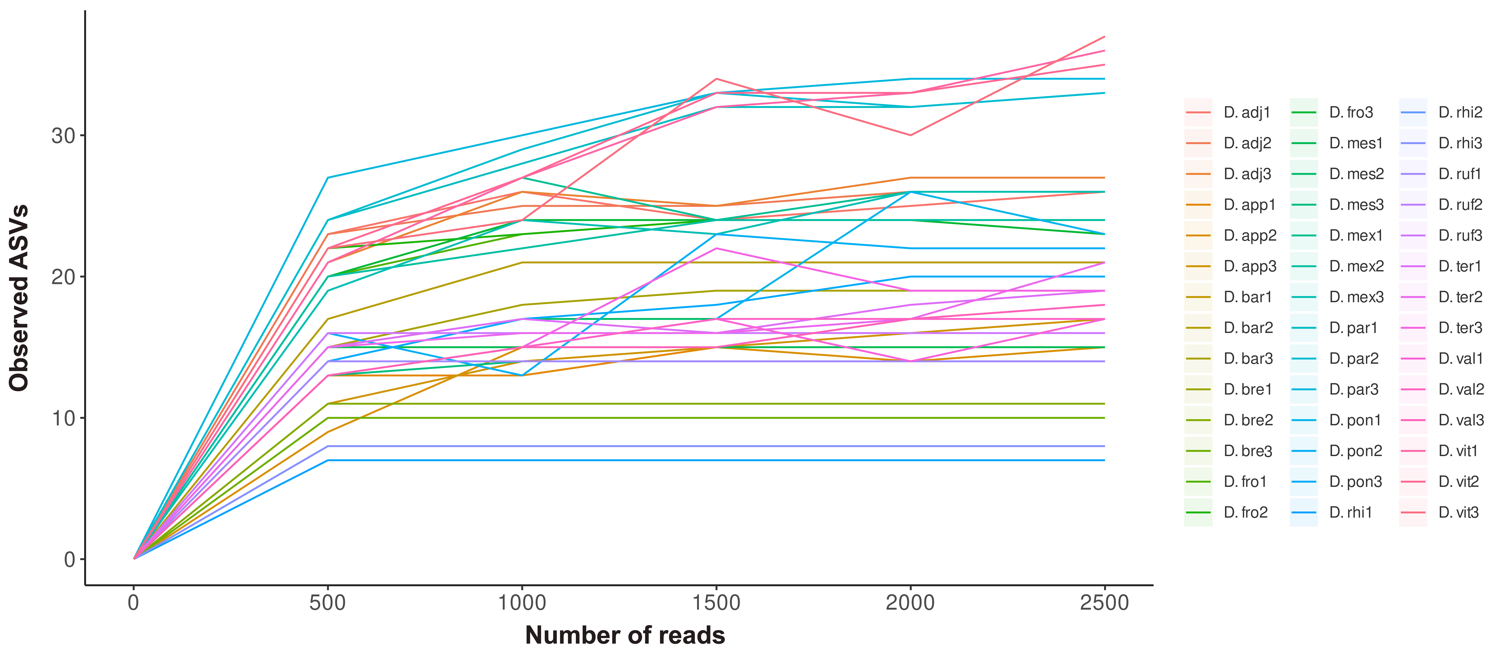
**

**Supplementary Figure 1.** Rarefaction curves of the libraries of fungal assemblages associated with the gut of 14 species of *Dendroctonus* genus.

**
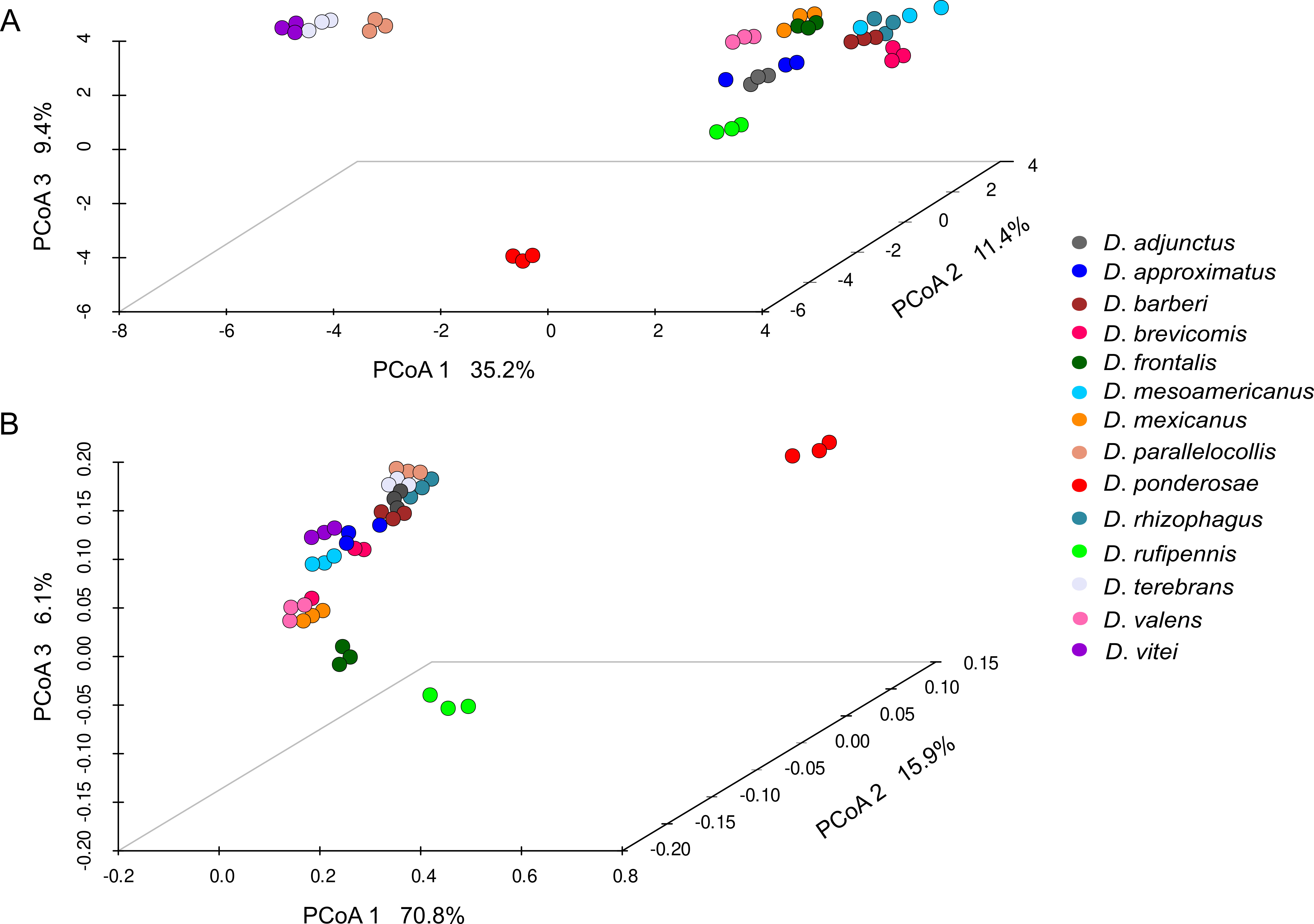
**

**Supplementary Figure 2.** Beta diversity analysis based on unweighted (A) and weighted (B) UniFrac distance matrices. The Principal Coordinate Analysis (PCoA) depicting variation in a multidimensional space of fungal assemblages from the gut of 14 species of *Dendroctonus,* dots of the same color represent biological replicates of the same bark beetle.

**
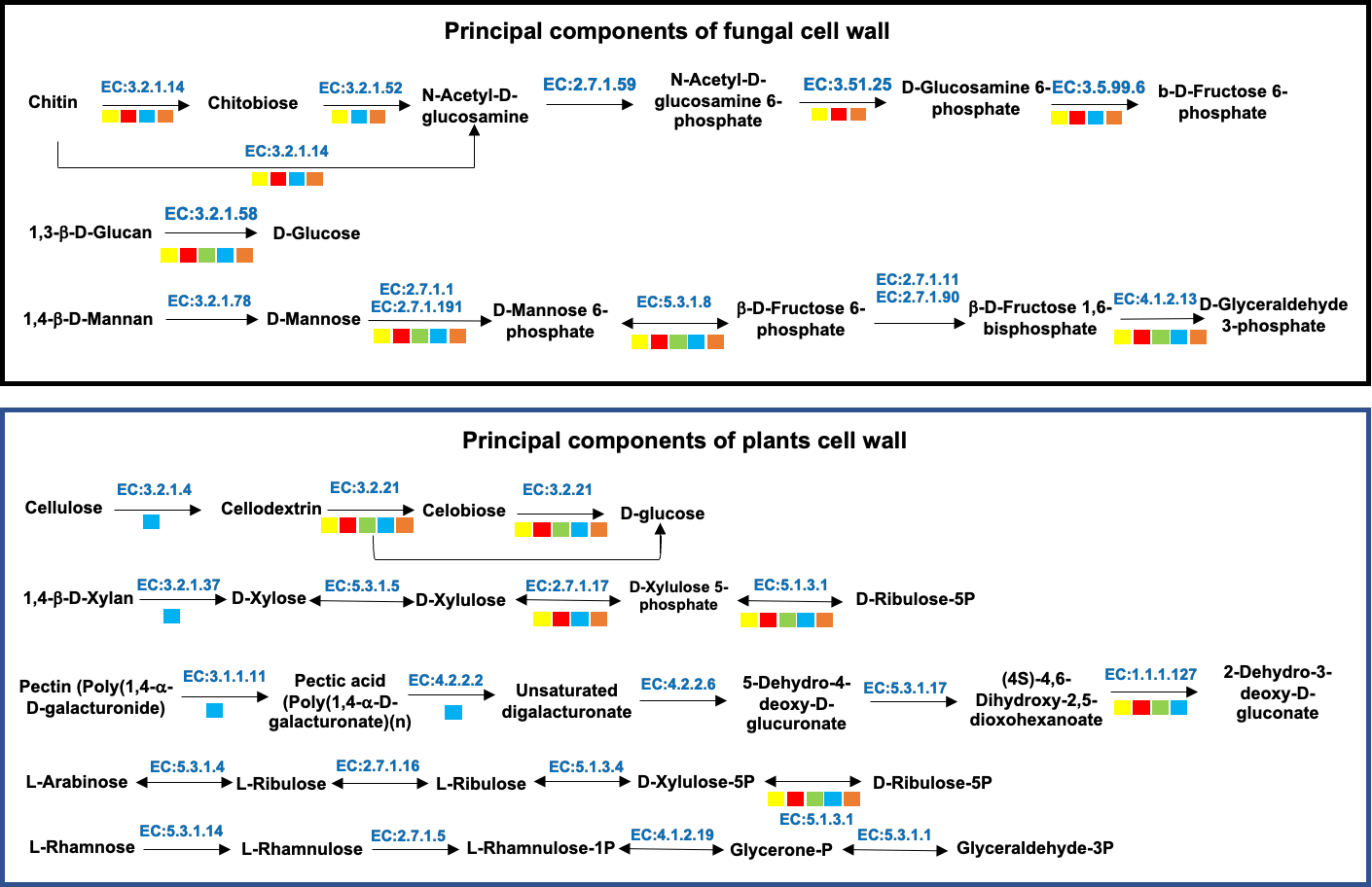
**

**Supplementary Figure 3.** Pathways for the degradation of fungal cell wall (Chitin, 1,3-β-Glucan, and 1,4-β- Mannan) and plant cell wall components (Cellulose, 1,4- β-D-Xylan, Pectin, L-Arabinose, and L- Rhamnose) by members to core mycobiome associated with bark beetles. Genomes used for the construction of metabolic networks. *Candida arabinofermentas* (GCA_001661425), *C. gorgasii* (ASM370898v3), *C. parapsilosis* (GCF_000182765), *C*. *ponderosae* (ASM370675v2), *C*. *railenensis* (GCA_935541525), *Kuraishia capsulata* (GCA_000576695), *K*. *molischiana* (GCA_024271875), *Nakazawaea ambrosiae* (GCA_024271865), *N*. *holstii* (GCA_003707025), *Ogataea pini* (GCA_003707665), *Yamadazyma scolyti* (GCA_003707025), *Y*. *tenuis* (GCA_000223465) and *Cladosporium cladosporioides* (GCA_002901145).  *Candida; Nakazawaea; Ogataea;*  *Clasdosporium* and *Yamadazyma*.
